# Communication Confidence, Aphasia Severity and Well‐Being: A Cross‐Sectional and Longitudinal Study in People With Post‐Stroke Aphasia

**DOI:** 10.1111/1460-6984.70316

**Published:** 2026-09-10

**Authors:** Sarah S. AlFraih, Emma Patchwood, Ciarán Kenny, Paul Conroy

**Affiliations:** ^1^ Speech‐Language Pathology and Audiology Department, College of Applied Medical Sciences King Saud bin Abdulaziz University for Health Sciences Riyadh Saudi Arabia; ^2^ King Abdullah International Medical Research Centre Riyadh Saudi Arabia; ^3^ Ministry of the National Guard – Health Affairs Riyadh Saudi Arabia; ^4^ Division of Psychology, Communication & Human Neuroscience The University of Manchester Manchester UK; ^5^ Division of Psychology and Mental Health University of Manchester Manchester UK; ^6^ School of Linguistic, Speech & Communication Sciences Trinity College Dublin Dublin Ireland

**Keywords:** chronic aphasia, communication confidence, confidence after stroke, longitudinal, well‐being

## Abstract

**Background:**

Communication confidence is increasingly recognised as a clinically crucial yet under‐researched topic in aphasiology. It has been reported to be one of the most valuable treatment outcomes by stroke survivors. A scoping review of therapies targeting confidence in stroke survivors revealed a growing interest in this topic and identified outcome measures for confidence. However, there were limited studies, with small sample sizes, that explored communication confidence in stroke survivors with aphasia. Further evidence is needed regarding this topic.

**Aims:**

This study aims to examine communication confidence in a comparatively larger and heterogeneous sample of people with post‐stroke chronic aphasia. The research questions explore (1) relationships between measures of language, communication confidence, confidence after stroke and well‐being and (2) stability of these measures over time and if changes align with self‐perceptions.

**Methodology:**

Participants with chronic aphasia after stroke (i.e., 6 months or more) and interested in exploring their communication confidence were recruited to participate in online assessments. Assessments included a test‐battery consisting of the ‘Western Aphasia Battery‐Revised–WAB‐R’, ‘Confidence after Stroke Measure‐CaSM’, ‘Communication Confidence Rating Scale for Aphasia‐CCRSA’ and ‘ONS‐4’. The test battery was administered at two separate time‐points approximately 6‐months apart.

**Results:**

Twenty‐seven participants were recruited for initial assessment and 18 were followed‐up after 6 months. Aphasia severities varied from mild to very severe with a mean WAB‐R aphasia quotient (AQ) of 74.4 (range from 9.1 to 98.2). Baseline measures indicated mean scores of 43.4/81 for the CaSM, 28.1/40 for the CCRSA and 25.9/40 for the ONS‐4. Statistically significant correlations were found between confidence after stroke and well‐being (*r* = 0.76, *p* < 0.001), communication confidence and confidence after stroke (*r* = 0.51, *p* = 0.006), and communication confidence and language skills (*r*_s_ = 0.68, *p* < 0.001). Although language skills and communication confidence remained relatively stable over time, confidence after stroke (*r* = 0.61, *p* = 0.01) and well‐being (*r* = 0.55, *p* = 0.02) significantly improved.

**Conclusion:**

The findings suggested stronger correlations between closer conceptual constructs (e.g., language and communication confidence, confidence and well‐being). Improvements in confidence and well‐being may have potential implications for rehabilitation approaches and may support the development of more holistic interventions for people with aphasia. This study also demonstrated the feasibility of conducting online, longitudinal research on stroke survivors.

**WHAT THIS PAPER ADDS:**

*What is already known on the subject*

- Communication confidence is a clinically crucial and valued topic for stroke survivors with aphasia. Previous research has explored relationships between aphasia and communication confidence in small samples of people with mild anomic aphasia or very severe aphasia. However, specific relationships between communication confidence and language skills, confidence after stroke and well‐being in a wider range of aphasia types and severities are not yet well understood.

*What this paper adds to the existing knowledge*

- Conceptually closer constructs had stronger correlations: significant correlations between language and communication confidence, confidence after stroke and communication confidence as well as confidence after stroke and well‐being. Confidence and well‐being seem to have improved over time while language and communication confidence remained stable. These findings offer refined perspectives on communication confidence and what might serve as potential intervention targets in chronic stages of aphasia. The study also demonstrated the feasibility of conducting online, longitudinal research on stroke survivors.

*What are the potential or actual clinical implications for this work?*

- Targeting improved confidence and well‐being after stroke when language skills have become more stable opens new avenues for therapeutic interventions

## 1 Introduction

Confidence is broadly defined as an individual's belief in their ability to succeed in a particular action or situation (Bandura 1977). The perception of self‐confidence plays a role in shaping behaviour and outcomes in diverse areas of life as it influences how individuals approach challenges, solve problems, and engage with others (Stajkovic and Luthans 1998). Well‐being is typically defined as a multidimensional state encompassing emotional, psychological, and social aspects of life which reflect an individual's overall satisfaction and functioning (Ryff and Keyes 1995). Bandura's (1997) theory of self‐efficacy asserts that individuals with higher confidence in their abilities are more likely to work toward goals, face obstacles, and achieve successful outcomes, all of which contribute to higher levels of psychological wellbeing. Empirical work supports this pathway, with general self‐efficacy shown to be positively associated with well‐being outcomes across cultures and life domains (Luszczynska et al. 2005). Evidence also supports the reverse direction: positive emotional states broaden individuals' thought‐action repertoires and build enduring personal resources, including confidence in one's own abilities (Fredrickson 2001), and positive affect has been shown to precede and contribute to success across multiple life domains (Lyubomirsky et al. 2005). Taken together, research suggests that confidence and well‐being are interrelated in several ways, with confidence serving as both a precursor and product of enhanced wellbeing.

Strokes are one of the leading causes of long‐term disability worldwide that have profound effects not only on the physical health of individuals, but also on their psychological well‐being. Stroke survivors often experience long‐term personal and social challenges, including changes in life roles, isolation, and depression, which can affect their adjustment and quality of life. Low self‐confidence post‐stroke is linked to decreased participation in physical activities, activity limitations, decreased independence, higher rates of anxiety and depression, and lower motivation to engage in rehabilitation (Jones et al. 2008; Rudd et al. 2017). Stroke survivors also report decreased confidence after stroke (Intercollegiate Stroke Working Party 2023). Additionally, there are long term effects that range from personal challenges to social adjustments which stroke survivors need to adapt to after their stroke. Shift in life roles, isolation, and depression are only a few of the problems commonly encountered after stroke which impact confidence and wellbeing (Jones and Riazi 2011). The relationship between stroke and confidence is complex and influenced by multiple factors, including physical and cognitive impairments, psychological well‐being, and social reintegration.

Communication confidence, defined as the self‐assurance in conveying ideas meaningfully in different contexts (Tonello et al. 2018), is a key psychosocial domain related to life participation, autonomy, and self‐efficacy (Babbitt and Cherney 2010). Though less researched than general well‐being after stroke, communication confidence is highly valued by stroke survivors as a critical treatment outcome, enabling them to apply their rehabilitation gains outside of the clinical setting (Pollock et al. 2014). According to the Stroke Association's *State of the Nation* document, 73% of stroke survivors report low confidence (Stroke Association 2016) and about 3.5 million stroke survivors worldwide experience aphasia (NIHR 2023). Stroke survivors with aphasia report a lower quality of life compared to those without, affecting multiple aspects of their lives (Hilari 2011; Hilari et al. 2012).

In a computer‐based language treatment study, participants with aphasia reported a significant increase in communication confidence without any significant changes in language skills (Babbitt and Cherney 2010). This might indicate that communication confidence is a complex construct that does not necessarily have a direct‐linear relationship with degree of language impairment or symptom severity. Although it is possible for communication confidence and symptom severity to be aligned, such that individuals with more severe aphasia may report lower communication confidence compared to those with milder aphasia, this relationship can vary significantly on a personal level. For example, even minor impairments in communication can severely diminish confidence in highly skilled individuals, such as those whose professional lives involve public speaking. Conversely, others may maintain or even increase their communication confidence through successful participation in social interactions, even without substantial improvements in language skills. Additionally, there may be intermediary factors influencing communication confidence in stroke survivors. For example, a study which reported improvements in literacy skills and technology engagement in stroke survivors with aphasia reported a positive impact on confidence alongside literacy skills, without a primary focus on improving face‐to‐face communication (Cetinkaya et al. 2024), supporting the concept that successful participation positively feeds back and reinforces communication confidence (Tonello et al. 2018). Confidence and skill often interact in dynamic ways that vary from person to person which means that while symptom severity may impact confidence, other factors, such as personality, past experiences, and the perceived importance of communication, can further complicate this relationship (Robertson 2021).

A recent study examined the relationship between communication confidence and mild symptoms of aphasia (Jacobs and Ellis 2023). Findings revealed weaker correlations between measures of symptoms/ levels of impairment and communication confidence and stronger corelations with confidence and age and education in participants with mild anomic aphasia (*n* = 12). They concluded that interpersonal characteristics, such as age and education, may influence communication confidence in people with mild aphasia. These findings were similar to another study conducted on stroke survivors with severe aphasia where they concluded that self‐efficacy ratings are lower in people with aphasia and are not proportionate to the level of language impairment and more related to functional communication (Olsson et al. 2024). Another study reported that communication confidence is more strongly associated to life participation and cognition rather than aphasia severity (Chiou and Yu 2018). Although these studies provided valuable preliminary insights into the relationship of these concepts, some had relatively small sample sizes, while others focused on fewer subgroups of stroke survivors with aphasia, such as mild anomic aphasia or severe aphasia. This highlights the need for further research on this topic in a wider range of aphasia types and severities. Additionally, while aphasic symptoms may become relatively stable during the chronic phase post‐stroke, less is known about whether self‐perceptions of communication confidence, general confidence after stroke, and well‐being follow a similar pattern or remain more dynamic over time. Understanding the stability of these psychosocial constructs may help identify potential targets for intervention beyond language intervention alone.

The present study aims to further investigate these interactions by sampling various critical measures such as aphasia severity and symptoms, communication confidence, stroke‐related confidence and overall well‐being, in a range of stroke survivors with chronic aphasia. It aims to consider relationships between language skills and these behaviours and attitudes. In addition, the present study will explore the stability of aphasic symptoms and self‐reported views of confidence related measures and well‐being over time within the chronic phase post‐stroke.

Research questions:

1. How do measures of language, communication confidence, confidence after stroke and well‐being relate to each other, in a cross‐sectional sample of people with stroke‐related chronic aphasia?
2. Do these measures change over time within the chronic phase after stroke in the context of usual clinical care, and do these changes align with participants’ broader self‐perceived changes in communication confidence and well‐being?

## 2 Methods

### 2.1 Design

A cross‐sectional, longitudinal online study was conducted to address the research questions. The study has been reported according to the checklist for Strengthening the Reporting of Observational Studies in Epidemiology (STROBE) (Von Elm et al. 2007), the completed checklist can be found in Appendix A. Ethical approval was granted by the relevant Research Ethics Committee on 07/06/2023 (Ref: 2023‐15398‐29060).

### 2.2 Test‐Battery

The test battery for this study was partly informed by a scoping review (AlFraih et al. 2024), where we identified tools for quantifying confidence‐related constructs in stroke survivors, including communication confidence and confidence after stroke. Additional measures of aphasia symptoms and severity, as well as psychological well‐being, were selected based on standardised measures that were judged to best reflect the broader constructs of interest in this study. The test‐battery for the current study therefore consisted of four assessment tools. Modifications were made for online administration as needed, and the measures are detailed below:

‐ *Western Aphasia Battery‐Revised—WAB‐R (Kertesz* 2007 *)*: To assess aphasia type and severity, the Aphasia Quotient (AQ) and Language Quotient (LQ) were obtained as per the guidelines for a core outcome set for aphasia research (Wallace et al. 2019). Modifications and guidelines to the online administration of the WAB‐R were followed as set out by Dekhtyar et al. (2020).
‐ *Communication Confidence Rating Scale for Aphasia—CCRSA (Babbitt* et al. 2011 *)*: To explore communication confidence, this self‐rating questionnaire was administered utilising a 100‐point scale across 10 items reflecting different aspects of communication confidence. Scores were summed and converted to a total score out of 40 for analysis, with higher scores indicating greater communication confidence.
‐ *Confidence after Stroke Measure—CaSM (* *Horne* et al. 2017 *)*: This self‐rating questionnaire was used to explore self‐confidence, positive attitude and social confidence through a four‐point scale across 27 items, with a maximum score of 81. Higher scores indicate greater confidence after stroke.
‐ *ONS‐4 (ONS* 2018 *)*: This questionnaire was used to explore personal well‐being during the time of assessment through four self‐rating items assessing life satisfaction, sense of worthwhileness, happiness, and anxiety. These domains reflect evaluative and emotional dimensions of well‐being and are widely used to capture an individual's broader psychological well‐being. Each item is rated on an 11‐point scale, with a maximum total score of 40. Higher scores indicate better overall well‐being

The above test‐battery was administered online by the first author, a Speech and Language Therapist (SLT), at two time points, approximately 6–7 months apart, via video‐conferencing while participants were in their own homes. Standardised administration procedures were followed for each measure, with adaptations for online delivery where required. Based on recommendations from Patient and Public Involvement (PPI) advisors, the questionnaires were displayed on screen during administration to support accessibility. Each question was presented one at a time, with the wording shown on the screen and read aloud to participants. Adequate font size was used, alongside visual representations of the Likert scales, to facilitate comprehension and support participation for people with aphasia.

At follow‐up, in addition to re‐administering the test‐battery, participants were asked two additional questions to explore self‐perceived changes in communication confidence and communication‐related habits: **(1) “How would you describe your communication confidence since we last met?”** (response options: less, same, better), and **(2) “Have there been any communication‐related changes since we last met?”** If participants responded “yes” to the second question, they were invited to briefly describe these changes.

### 2.3 Patient and Public Involvement—(PPI)

PPI (Patient and Public Involvement) advisors were consulted in an iterative process throughout the study's design. Over 3–4 individual meetings, four stroke survivors provided input on study design, consent forms, participant information sheets, and assessment adaptations. This ensured the materials were accessible, informative, and appropriate for stroke survivors with aphasia accessing materials online. Each PPI advisor participated in at least three sessions, offering continuous feedback.

### 2.4 Participants

Individuals with chronic post‐stroke aphasia (i.e., 6‐months or more) were recruited if they scored within or above the 50th percentile for receptive language and 20th percentile for expressive language on the WAB‐R. These thresholds were selected to ensure participants had sufficient receptive and expressive language abilities to engage with the online assessment process, including understanding the self‐rating questionnaires and expressing their responses. This was particularly important given the online format and the self‐reflective nature of the confidence and well‐being measures. Eligible participants were those interested in exploring their communication confidence, willing and able to participate in online assessments, including sufficient English language to engage. Stroke survivors who informed us of a clinical diagnosis of depression or chronic anxiety, or whose aphasia was of a severity that would limit their engagement in the sessions, were excluded from the study. This was a functional rather than a strict severity‐based criterion.

### 2.5 Recruitment

Potential participants were approached through stroke community groups in Manchester and online through leaders of stroke groups across the UK. Interested participants were provided with study information sessions online and informed consent taken after any questions had been answered. Participants underwent two online assessments at two separate time‐points for this study: an initial baseline assessment (August—October 2023) and a follow‐up assessment after approximately 6–7 months (26–35 weeks). The target of 30 participants was selected pragmatically based on recruitment feasibility and to allow for a broad exploratory sample of individuals with chronic aphasia. Within this pool, it was planned to scope out the interest in participating in a bespoke communication‐confidence focused therapy study (Confident Communication with Aphasia—CoCA study). It was anticipated that approximately six participants would be recruited into the CoCA study, as this reflected the practical requirements of a small‐group therapy programme. This would leave an estimated 24 participants for the follow‐up longitudinal assessment, allowing for exploration of changes over time in the remaining cohort.

### 2.6 Data Analysis

The analysis of the data followed a quantitative approach to address the research questions. After checking the distributions of data to inform analysis, the exploration of relationships between constructs of communication confidence, aphasia severity, confidence after stroke and well‐being were completed. Additionally, changes over time and self‐perceptions of change were computed and compared.

All data were scored, computed and saved on Microsoft Excel then uploaded into SPSS for data analysis. Descriptive statistics were used to summarise demographics and baseline/follow‐up measures from the sample. Correlations between the test‐battery assessments were explored in addition to non‐parametric rank tests to explore stability of measures between baseline and follow‐up. All statistical analyses were performed using SPSS version 29 (IBM 2022).

## 3 Results

### 3.1 Participants and Descriptive Data

**FIGURE 1:**
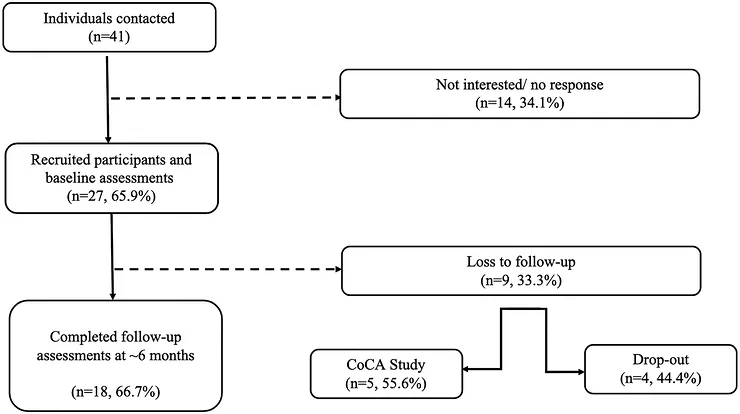
Flow diagram of individuals at each stage of the study.

Twenty‐seven participants with chronic aphasia after stroke were recruited and completed the initial assessment session (baseline). Five/27 (18.5%) participants were redirected after baseline to participate in the Confident Communication with Aphasia (CoCA) therapy study and 18/27 (66.7%) completed follow‐up assessments (Figure 1). The majority of participants were male (15/27, 55.6%). Ages ranged from 44 to 79 years old with a mean participant age of 60.7 years old (SD = 9.1). Participants’ time since stroke ranged from 6 to 363 months (30 years) with a mean of 94.7 months (7.8 years, SD = 85.9). Table 1 illustrates the demographics and clinical characteristics for the study participants. Participants’ aphasia severity was as measured by the WAB‐R (AQ). Participants’ aphasia severity ranged from mild (*n* = 16, 59.2%) to moderate (*n* = 9, 33.3%) and very severe (*n* = 2,7.4%) aphasia. Coincidentally, no participants fell within the severe aphasia WAB‐R range, although the sample did span the full range of aphasia severities (mild to very severe). Aphasia types determined by WAB‐R AQ, also varied from Anomic (*n* = 14, 51.8%), Conduction (*n* = 4, 14.8%), Broca's (*n* = 8, 29.6%) and Wernicke's (*n* = 1, 3.7%).

**TABLE 1:** Participants’ demographics and clinical data at baseline (*N* = 27).

Participant ID	Age	Gender	Months since stroke	Aphasia classification^a^	Aphasia severity^a^
**1**	64	Female	91	Anomic	Mild
**2**	58	Female	105	Anomic	Moderate
**3**	77	Male	19	Conduction	Moderate
**4**	73	Male	23	Wernicke's	Moderate
**5**	62	Male	124	Broca's	Very Severe
**6**	69	Male	155	Anomic	Mild
**7**	56	Female	113	Anomic	Mild
**8**	66	Male	82	Anomic	Mild
**9**	60	Female	242	Conduction	Mild
**10**	54	Female	124	Broca's	Moderate
**11**	54	Female	69	Conduction	Mild
**12**	49	Female	9	Broca's	Moderate
**13**	79	Male	58	Anomic	Mild
**14**	58	Male	6	Anomic	Mild
**15**	69	Male	22	Anomic	Mild
**16**	57	Female	45	Anomic	Mild
**17**	64	Female	6	Anomic	Mild
**18**	44	Male	99	Broca's	Moderate
**19**	56	Male	101	Anomic	Mild
**20**	66	Female	363	Anomic	Mild
**21**	60	Male	169	Broca's	Very Severe
**22**	59	Female	93	Broca's	Moderate
**23**	60	Male	268	Anomic	Mild
**24**	74	Male	46	Anomic	Mild
**25**	62	Male	77	Broca's	Moderate
**26**	47	Male	39	Conduction	Mild
**27**	44	Female	9	Broca's	Moderate
**Mean (SD)**	60.7 (9.1)	—	94.7 (85.9)	—	—
**Range**	44‐79	—	6‐363	—	—

^a^Aphasia classification and severity determined based on WAB‐R scores, that is, 0–25 very severe, 26–50 severe, 51–75 moderate and 76 and above is considered mild.

### 3.2 Assessment Measures Outcome Data

Detailed results of the test‐battery at the initial baseline assessment time‐point and follow‐up assessment time point can be found in Appendix B.

### 3.3 Relationships Between Assessment Measures at Baseline

The first research question focused on the relationships between the study's assessment measures for language, communication confidence, confidence after stroke and well‐being. Table 2 below illustrates the results of the correlations conducted for each pair of measures.

**TABLE 2:** Correlations between assessment measures.

Constructs and assessment measures		Correlation coefficient	*p* value	Correlation strength
Communication Confidence *(CCRSA)*	Language *(WAB‐R (AQ))*	*r_s_* (25) = 0.68	<0.001*	Strong correlation
Communication Confidence *(CCRSA)*	Confidence after Stroke *(CaSM)*	*r*(25) = 0.51	0.006**	Moderate correlation
Communication Confidence *(CCRSA)*	Well‐being *(ONS‐4)*	*r*(25) = 0.33	0.092	Weak correlation
Confidence after Stroke *(CaSM)*	Language *(WAB‐R (AQ))*	*r_s_* (25) = 0.21	0.299	Weak correlation
Confidence after Stroke *(CaSM)*	Well‐being *(ONS‐4)*	*r*(25) = 0.76	<0.001*	Strong correlation
Language *(WAB‐R (AQ))*	Well‐being *(ONS‐4)*	*r_s_* (25) = 0.15	0.455	Very weak correlation

*Note*: *r* = Pearson's correlation coefficient; *r_s_* = Spearman's rank correlation coefficient. Correlation strength was interpreted according to Evans 1996, 0.00–0.19 = very weak, 0.20–0.39 = weak, 0.40–0.59 = moderate, 0.60–0.79 = strong, and 0.80–1.00 = very strong. *p* < 0.05, *p* < 0.01.

In summary within the selected assessment measures, three correlations appeared to be statistically significant at baseline which were (1) language skills and communication confidence, (2) communication confidence and confidence after stroke and (3) confidence after stroke and wellbeing. Of these, the correlations between language skills and communication confidence, and between confidence after stroke and well‐being, were strong, while the correlation between communication confidence and confidence after stroke was moderate.

### 3.4 Description of Changes in Measures Over Time

The assessments included in the study test‐battery at baseline were re‐administered during follow‐up. As outlined above, five participants were removed from follow up after being recruited to a communication confidence focused therapy study (CoCA). This left a maximum possible follow‐up cohort of 22 participants for longitudinal re‐assessment, of which, 18 were successfully re‐assessed. There was a 55.6% (10/18) success rate in achieving follow‐up within the planned 26–28 weeks from baseline, the remaining 44.4% of participants (8/18) exceeded this timeframe by 1–7 weeks, resulting in an overall follow‐up period of 26–35 weeks. The four participants who did not complete follow‐up were either unavailable during the follow‐up period or did not respond to contact attempts. Despite participant attrition, the observed changes in each measure were explored. Changes in raw scores for each measure of the study's test‐battery is illustrated in Table 3 in addition to participants’ responses to changes in communication confidence *“How would you describe your communication confidence since we last met?”* and communication habits *“Have there been any communication‐related changes since we last met?”* and a description of reported changes.

**TABLE 3:** Changes in scores between baseline and follow‐up based on raw scores (*N* = 18) and self‐reports of changes in communication confidence.

Participant ID	WAB‐R (AQ)	CCRSA	CaSM	ONS‐4	Description of communication confidence since baseline	Alignment of self‐perceptions and CCRSA scores	Communication‐related changes since baseline?	Description of changes
**1**	+1	−1	+7	0	Same	No	No	
**3**	+3.2	+6	−2	+3	Less	No	Yes	Participation in research study
**4**	−1.7	+3	+4	+6	Same	No	No	
**5**	NA	−2	+3	−9	Same	No	No	
**6**	−10.1	0	+5	+4	Better	No	No	
**7**	+3.2	+2	+9	+8	Less	No	No	
**8**	−2.4	+1	0	+2	NA	NA	NA	
**9**	+1.9	−3	+2	0	Same	No	No	
**12**	+5.8	+7	+13	−2	Better	Yes	Yes	Started SLT
**13**	+1.7	−4	−3	0	Same	No	No	
**14**	+7.1	+2	+2	+6	Less	No	Yes	Started writing therapy
**15**	−0.3	−5	+11	+5	Better	No	No	
**16**	+1.2	+4	+2	+8	Less	No	Yes	Stopped SLT
**17**	+2.4	+6	+11	+11	Better	Yes	No	
**20**	+8.1	+5	−6	−3	Same	No	No	
**23**	−1.2	+3	+1	+5	Same	No	No	
**24**	+6.8	0	+3	+4	Same	Yes	Yes	Participation in research study
**25**	−2.8	0	+6	+6	Better	No	Yes	Participation in research study

*Note*: + = increase, — = decrease, 0 = no change, NA = missing data. Shaded cells in the “Alignment of self‐perceptions and CCRSA scores” column indicate cases where participants’ self‐perceived communication confidence aligned with the direction of change observed in their CCRSA scores.

### 3.5 Perceptions of Changes in Communication Confidence

During follow‐up assessment, participants were asked if they perceived any changes in their levels of communication confidence relative to the baseline assessment (response options: same/better/less). Additionally, they were also asked if any support for communication‐related changes were worth noting (such as starting or stopping speech and language therapy for example). Their responses were compared to their CCRSA scores at both assessment time points. Table 3 illustrates the amount of change in participants' raw scores, responses to follow‐up questions and the alignment of self‐perceptions on communication confidence and actual change in CCRSA scores. The results seem to show some discrepancies between self‐perceptions on changes in communication confidence and communication confidence scores based on the CCRSA. Of the 18 participants who completed follow‐up, 17 provided responses to the self‐perception questions regarding communication confidence and communication‐related changes. For example, all participants who reported that they felt they had less communication confidence during the follow‐up assessment scored higher on the CCRSA during follow‐up (4/17) with a 2–6 points increase. Half of the participants who reported having the same levels of communication confidence (4/8) scored 1–4 points lower on the CCRSA during follow‐up, while (3/8) scored 3–5 points higher, and 1/8 showed no change in CCRSA score. Overall, only three participants had an agreement between their self‐perception of changes in their communication confidence and CCRSA scores.

Table 4 below illustrates the results of the Wilcoxon signed‐rank tests conducted for each measure at baseline and follow‐up. The CaSM and ONS‐4 showed differences that were statistically significant which indicated that measures of confidence after stroke and well‐being in our participants increased over the period of approximately 6 months.

**TABLE 4:** Wilcoxon signed‐rank results for assessment measures.

Assessment measure	Baseline				Follow‐up				Wilcoxon Signed‐Rank		
	M	SD	*Mdn*	*Q1–Q3*	*M*	*SD*	*Mdn*	*Q1‐Q3*	*W*	*p*	*r*
**WAB‐R (AQ)**	81.5	14.3	87.4	79.3–91.8	82.9	14.8	87.6	79.0–93.7	45.5	0.142	0.36
**CCRSA**	28.8	5.1	29.5	24.5–31.8	30.1	4.5	29.5	26.0–33.5	33.5	0.131	0.36
**CaSM**	44.7	9.5	44.0	39.5–52.3	48.5	8.5	48.5	43.5–52.8	22.0	0.010^*^	0.61
**ONS‐4**	26.7	6.2	25.0	24.0–31.0	29.7	6.0	30.0	25.3–34.8	19.0	0.020^*^	0.55

*Notes*: Sample sizes reflect participants with complete baseline and follow‐up data for that measure: *n* = 17 for WAB‐R (AQ); *n* = 18 for CCRSA, CaSM, and ONS‐4 (one additional participant had follow‐up data for these three measures but not for the WAB‐R). M = mean; SD = standard deviation; Mdn = median; Q1 = first quartile (25th percentile); Q3 = third quartile (75th percentile); W = Wilcoxon signed‐rank test statistic; *p* = *p* value for significance; *r* = effect size (*r* = Z/√N).

*Statistically significant at *p* < 0.05.

## 4 Discussion

This study explored relationships between communication confidence, language, confidence after stroke and well‐being in stroke survivors with chronic aphasia (6 months or more) as well as the exploration of the stability of these constructs over time, and how self‐perceived change aligned with measured change. Correlational analyses, which cannot establish causation, suggested there were some strong as well as weaker statistically significant associations between the measures. Specifically, the strongest correlational relationship was between confidence after stroke and well‐being, confirming that an individual with high general (i.e., not specifically communication‐focused) confidence after stroke was likely to also report relative high levels of well‐being. Similarly, aphasia severity and communication confidence showed strong positive associations, that is, the milder one's aphasic symptoms, the more likely an individual was to report more communication confidence. In between these strong correlations was the weaker though significant one between communication confidence and confidence after stroke. This interesting pattern of correlational relationships that arise in our study may be best described as an adjacency effect. We noticed that correlations are strongest between related measures that are conceptually adjacent or neighbouring, while measures further apart show weaker or non‐significant relationships. In other words, the WAB‐R and CCRSA are conceptually closer (as both are directly related to communication), so their correlation is stronger and statistically significant. The CCRSA and the CaSM are also conceptually related, as both deal with aspects of confidence, potentially explaining the significant correlation between them. We conceptualised this pattern as a chain‐like relationship, in which the four constructs are ordered along a continuum from narrow and domain‐specific to broad and generalised: language ability (WAB‐R)—communication confidence (CCRSA)—general confidence after stroke (CaSM)—general well‐being (ONS‐4). Within this chain, constructs that are conceptually closer to one another (i.e., adjacent links) showed stronger, statistically significant correlations, while constructs further apart in the chain showed progressively weaker, non‐significant correlations. This is illustrated by the strong correlation between confidence after stroke (CaSM) and well‐being (ONS‐4), which sit adjacent to one another at the broader end of the chain, in contrast to the weaker, non‐significant correlation between communication confidence and well‐being (CCRSA and ONS‐4), which are separated by one additional link. This may indicate that adjacency in the chain reflects conceptual specificity and breadth rather than superficial thematic similarity. Although CCRSA and CaSM are both on the surface, “confidence” measures and were themselves significantly correlated, only the CaSM occupied the link directly adjacent to well‐being, which accounts for the discrepancy between the two measures’ respective correlations with the ONS‐4.

An interesting finding in our sample, containing a wider variety of aphasia severities, is the somewhat linearity between impairment and impact that our data suggests, whereas studies on stroke survivors with mild anomic aphasia report non‐linear relationships (Jacobs and Ellis 2023). In addition to the studies that conclude that although self‐efficacy or confidence may be lower in individuals with severe aphasia, it does not necessarily align with the level of language impairment (Olsson et al. 2024). Previous studies also report that communication confidence in people with aphasia is more related to life participation and cognition rather than language ability (Chiou and Yu 2018).

Although the aphasia severity measures and communication confidence measures were relatively stable over this time window of approximately six to seven months for the group, the confidence after stroke and well‐being measures showed positive change which reached statistical significance. Our data suggest, for the reduced cohort of *n* = 18, many participants reported feeling enhanced general confidence and well‐being despite only four participants starting new language‐related interventions during this time. A possible explanation for this is that these broader constructs may remain malleable long after language skills have stabilised. Although the WAB‐R and CCRSA are more directly linked to the impairment itself, confidence after stroke and well‐being seem to be shaped by ongoing lived experience, such as gradually mastering everyday communication situations, re‐engaging in valued social roles and renegotiating one's identity as part of adjusting to life with chronic aphasia (Bandura 1997; Shadden 2005; Grohn et al. 2014). From this perspective, positive change in these measures does not necessarily require formal intervention. It is also possible that taking part in the study itself contributed to these gains, as the regular contact with the research team and being asked to reflect on one's own communication may have acted as a mild activating experience, similar to what has been described as a Hawthorne effect (McCambridge et al. 2014). However, these interpretations should be weighed against other, less substantive explanations. No self‐report measure is free of measurement error, and in the absence of established Minimally Important Clinical Differences for the CaSM and ONS‐4 (Jaeschke et al. 1989), we cannot confirm that the observed changes exceed what might be expected from the measures' error margins alone. Regression to the mean (Barnett et al. 2005), response shift when re‐rating subjective constructs (Sprangers and Schwartz 1999) and selective attrition, whereby the reduced follow‐up cohort may have been the more engaged or better‐adjusted portion of the original sample, also remain possible counter‐explanations that cannot be ruled out without a control group. These findings should therefore be interpreted cautiously and may represent hypothesis‐generating rather than confirmatory evidence of naturally occurring change.

Self‐perceptions of communication confidence did not consistently align with changes in CCRSA scores. This divergence may reflect the complexity of measuring communication confidence, but it may also be explained by differences in the nature and validity of the two approaches used. The follow‐up question asked participants to retrospectively evaluate perceived change, whereas the CCRSA provided a structured self‐rating of communication confidence at each time point. Discrepancies may therefore have been influenced by recall bias, response shift, measurement error, or by participants drawing on broader lived experiences of communication that were not fully captured by the CCRSA. These findings should be interpreted cautiously and explored further in future research.

It is worth noting that a group of participants (*n* = 5) opted for a communication confidence‐focused therapy after baseline and were not included within the follow‐up dataset. Although only some participants were offered, due to range of severity considerations, those who were not interested in taking part for either practical or personal reasons were included in the follow‐up dataset which may have served as a potential confound. This has been previously reflected in the literature in a study that examined the impact of readiness for treatment in inpatients with depression, findings suggest that higher levels of readiness for treatment, confidence and motivation before commencing therapy targeting depression were associated with better outcomes (McCarthy et al. 2024). It is also important to acknowledge that other factors, such as education levels, marital/familial relationships and availability of support networks, and opportunities to practice functional communication, were not measured and are likely to have influenced confidence levels. These unmeasured factors could have accounted for some of the variance in outcomes as reported in the findings from previous studies conducted on homogeneous samples of stroke survivors with mild anomic aphasia (Jacobs and Ellis 2023) while other studies with wider samples of aphasia severities report no influence on communication activity from these factors (Mazaux et al. 2013). Although the available data provide valuable insights, the absence of these additional predictors represent a limitation of the study. It is noted that although our study sample, by coincidence, did not contain any individuals within the WAB‐R severe aphasia classification, it spanned severities from mild to very severe, with a sample size comparable to the larger of the previous studies focused specifically on communication confidence in people with aphasia (e.g., Chiou and Yu 2018). In comparison with previous prospective studies that have administered the CCRSA directly to people with aphasia, which have typically involved small cohorts (e.g., *n* = 12 in Jacobs and Ellis 2023; *n* = 10 in Carr et al. 2022), this study features a relatively large sample for a longitudinal design of this kind, given the recruitment, assessment‐burden and attrition challenges inherent in repeated testing of stroke survivors with aphasia. However, the sample size may still not be sufficient to represent the broader population of stroke survivors with chronic aphasia (Brady et al. 2014; Kristinsson et al. 2022). Additionally, the wide range of months post stroke at baseline may potentially be confounding and would arguably be stronger if participants were recruited within the first 6–12 months post stroke. Due to recruitment challenges, we had to be as inclusive as possible and did not have an upper limit cutoff for time since stroke. Also, for practical reasons it was not possible to include the full spectrum of severity of people with aphasia. Although the exclusion criteria of those who informed us of a clinical diagnosis of depression or chronic anxiety was in an effort to prevent potential distress that may arise from the study, it further limited our sample and limited its true representation.

However, this study offers further insights into the factors that influence the clinically important construct of communication confidence for people living with chronic aphasia after a stroke. This exploratory study expands on the concept of communication confidence as it identifies potential relationships with language, confidence after stroke and well‐being. It also provides insights into measuring this construct that may be further explored in future research with larger sample sizes and more robust follow‐up data. Specifically, the discrepancies observed between changes in CCRSA scores and participants' self‐reported perceptions of change suggest that scale scores and subjective perceptions are not interchangeable, and that pairing standardised measures with direct self‐perception questions may offer a fuller picture of communication confidence. The pattern of correlations also suggests that the CCRSA captures a construct distinct from both aphasia severity and general confidence after stroke, supporting its use alongside, rather than in place of, broader psychosocial measures. Finally, the relative stability of CCRSA scores over approximately six months offers preliminary evidence of the measure's stability in the chronic phase, though the absence of established Minimally Important Clinical Differences for these measures remains a priority for future measurement research. Together, these findings reinforce the multifaceted nature of communication confidence in chronic aphasia and its influence by factors beyond aphasia severity, including self‐confidence, social‐confidence and attitude based on the domains explored in the CaSM.

Since communication confidence is affected by multiple, interrelated factors, it requires a more nuanced and comprehensive approach to both evaluation and intervention. This includes addressing psychological and social aspects alongside language therapy (Manning et al. 2021; Northcott et al. 2017). Tailoring therapies to address the broader spectrum of factors influencing communication confidence—such as aphasia severity, well‐being, and social interactions—may lead to more effective and sustained outcomes (James Lind Alliance 2025). Taking into consideration a more holistic view of patients' experiences and the incorporation of a variety of therapeutic modalities, like group therapy and mindfulness, may be beneficial (NICE 2023). Given the stability of some correlations over time, the results may also suggest that building confidence can have dual benefits: improving communication confidence and enhancing overall well‐being. This dual impact makes confidence‐building an attractive target for intervention. By focusing on boosting self‐efficacy and confidence, clinicians may be able to foster improvements not only in communication but also in the patient's emotional and psychological health.

Additionally, this study demonstrates the feasibility of conducting online, longitudinal research on stroke survivors, though attrition remains a challenge for maintaining statistical power. The online nature of this study allowed us to reach a wider pool of participants, not just limiting us to a specific geographical region. Due to the online nature, adaptations were made to the assessment materials to be suitable for online administration. Despite the wider reach, the online format may have introduced new barriers such as technical difficulties and might have also limited our participant pool to those with internet access and already familiar with video‐conferencing (Tyagi et al. 2018). It is also acknowledged that the aspect of self‐referral into research studies may have presented a selection bias toward those with higher confidence levels, which may skew the results and be unrepresentative of the true population. This selection bias has been reported in a systematic review of various Intensive Comprehensive Aphasia Program (ICAP) studies due to their recruitment methods (Monnelly et al. 2024).

## 5 Conclusion

This study underscores the complexity of communication confidence in individuals with chronic aphasia and highlights the need for further research in this area. Significant relationships were found between aphasia severity, communication confidence, post‐stroke confidence, and psychological well‐being. The study also demonstrated the feasibility of conducting online, longitudinal research on stroke survivors, though attrition remains a challenge for maintaining statistical power.

The findings suggest that while communication confidence and aphasia severity may remain stable over time, general confidence after stroke and well‐being can improve significantly, which may have important implications for rehabilitation strategies.

## Funding

This research was part of a PhD scholarship awarded to the first author by King Saud bin Abdulaziz University for Health Sciences through the Saudi Arabian Cultural Bureau in London.

## Data Availability

The data that supports the findings of this study are available in the supplementary material of this article.

## Appendix A Observational studies in epidemiology (STROBE) checklist.

### A.1

**Table:**

	Item No	Recommendation	Page No
Title and abstract	1	(*a*) Indicate the study's design with a commonly used term in the title or the abstract	1
		(*b*) Provide in the abstract an informative and balanced summary of what was done and what was found	1
**Introduction**			
Background/rationale	2	Explain the scientific background and rationale for the investigation being reported	2–3
Objectives	3	State specific objectives, including any prespecified hypotheses	3
**Methods **			
Study design	4	Present key elements of study design early in the paper	3
Setting	5	Describe the setting, locations, and relevant dates, including periods of recruitment, exposure, follow‐up, and data collection	4
Participants	6	(*a*) Give the eligibility criteria, and the sources and methods of selection of participants. Describe methods of follow‐up	4
		(*b*) For matched studies, give matching criteria and number of exposed and unexposed	NA
Variables	7	Clearly define all outcomes, exposures, predictors, potential confounders, and effect modifiers. Give diagnostic criteria, if applicable	3–4
Data sources/ measurement	8*	For each variable of interest, give sources of data and details of methods of assessment (measurement). Describe comparability of assessment methods if there is more than one group	3–4
Bias	9	Describe any efforts to address potential sources of bias	4, 9—10
Study size	10	Explain how the study size was arrived at	4
Quantitative variables	11	Explain how quantitative variables were handled in the analyses. If applicable, describe which groupings were chosen and why	4–5
Statistical methods	12	(*a*) Describe all statistical methods, including those used to control for confounding	4–5
		(*b*) Describe any methods used to examine subgroups and interactions	5–7
		(*c*) Explain how missing data were addressed	5–6
		(*d*) If applicable, explain how loss to follow‐up was addressed	5–6, 9–10
		(*e*) Describe any sensitivity analyses	NA
**Results **			
Participants	13*	(a) Report numbers of individuals at each stage of study, for example, numbers potentially eligible, examined for eligibility, confirmed eligible, included in the study, completing follow‐up, and analysed	5–6
		(b) Give reasons for non‐participation at each stage	5–6
		(c) Consider use of a flow diagram	5
Descriptive data	14*	(a) Give characteristics of study participants (e.g., demographic, clinical, social) and information on exposures and potential confounders	5–6, 14–15
		(b) Indicate number of participants with missing data for each variable of interest	14–15
		(c) Summarise follow‐up time (e.g., average and total amount)	5–6
Outcome data	15*	Report numbers of outcome events or summary measures over time	5–9, 14–15
Main results	16	(*a*) Give unadjusted estimates and, if applicable, confounder‐adjusted estimates and their precision (e.g., 95% confidence interval). Make clear which confounders were adjusted for and why they were included	5–9
		(*b*) Report category boundaries when continuous variables were categorized	NA
		(*c*) If relevant, consider translating estimates of relative risk into absolute risk for a meaningful time period	NA
Other analyses	17	Report other analyses done, for example, analyses of subgroups and interactions, and sensitivity analyses	6–9
**Discussion **			
Key results	18	Summarise key results with reference to study objectives	7, 9–10
Limitations	19	Discuss limitations of the study, taking into account sources of potential bias or imprecision. Discuss both direction and magnitude of any potential bias	9–10
Interpretation	20	Give a cautious overall interpretation of results considering objectives, limitations, multiplicity of analyses, results from similar studies, and other relevant evidence	7–10
Generalisability	21	Discuss the generalisability (external validity) of the study results	10
**Other information **			
Funding	22	Give the source of funding and the role of the funders for the present study and, if applicable, for the original study on which the present article is based	10

## Appendix B Participants test‐battery measures.

### B.1

**Table:**

Participant ID	Baseline measures				Follow‐up measures			
	WAB‐R (AQ)	CCRSA	CaSM	ONS‐4	WAB‐R (AQ)	CCRSA	CaSM	ONS‐4
1	92.6	31	38	26	93.6	30	45	26
2	75.0	29	39	21	—	—	—	—
3	59.6	23	50	32	62.8	29	48	35
4	63.6	23	41	22	61.9	26	45	28
5	10.8	24	56	36	—	22	59	27
6	90.2	32	54	32	80.1	32	59	36
7	91.6	29	29	24	94.8	31	38	32
8	93.3	35	53	35	90.9	36	53	37
9	79.7	32	49	25	81.6	29	51	25
10	69.2	28	24	10	—	—	—	—
11	83.9	17	41	25	—	—	—	—
12	57.1	22	25	21	62.9	29	38	19
13	98.2	40	63	40	99.9	36	60	40
14	89.9	34	47	19	97.0	36	49	25
15	79.3	30	53	25	79.0	25	64	30
16	91.8	30	45	24	93.0	34	47	32
17	92.2	31	41	24	94.6	37	52	35
18	66.4	32	69	38	—	—	—	—
19	94.2	34	48	27	—	—	—	—
20	79.5	21	39	26	87.6	26	33	23
21	9.1	18	27	23	—	—	—	—
22	62.9	22	40	21	—	—	—	—
23	87.4	29	42	25	86.2	32	43	30
24	86.9	26	37	16	93.7	26	40	20
25	53.4	26	43	28	50.6	26	49	34
26	77.4	32	48	25	—	—	—	—
27	74.8	29	31	29	—	—	—	—
**Range**	9.1‐98.2	17‐40	24‐69	10‐40	50.6‐99.9	22‐37	33‐64	19‐40
**Mean**	74.4	28.1	43.4	25.9	83.0	30.1	48.5	29.7
**SD**	22.5	5.5	10.9	6.6	14.8	4.5	8.5	6.0
**Min and max overall score**	0–100	10–40	0–81	0–40	0–100	10–40	0–81	0–40

**N.B**. for the WAB‐R (AQ), lower scores indicate greater aphasia severity. For the CCRSA, lower scores indicate lower communication confidence. For the CaSM, lower scores indicate lower confidence after stroke. For the ONS‐4, lower scores indicate poorer well‐being.
